# Increased brain iron in patients with thyroid-associated ophthalmopathy: a whole-brain analysis

**DOI:** 10.3389/fendo.2023.1268279

**Published:** 2023-11-16

**Authors:** Hao Hu, Jiang Zhou, Wei Fang, Huan-Huan Chen, Wen-Hao Jiang, Xiong-Ying Pu, Xiao-Quan Xu, Wen-Hao Gu, Fei-Yun Wu

**Affiliations:** ^1^ Department of Radiology, The First Affiliated Hospital of Nanjing Medical University, Nanjing, China; ^2^ Department of Radiology, Taicang Affiliated Hospital of Soochow University, The First People’s Hospital of Taicang, Taicang, China; ^3^ Department of Endocrinology, The First Affiliated Hospital of Nanjing Medical University, Nanjing, China

**Keywords:** brain iron deposition, magnetic resonance imaging, quantitative susceptibility mapping, deep gray matter, thyroid-associated ophthalmopathy

## Abstract

**Background:**

To investigate the whole-brain iron deposition alternations in patients with thyroid-associated ophthalmopathy (TAO) using quantitative susceptibility mapping (QSM).

**Methods:**

Forty-eight patients with TAO and 33 healthy controls (HCs) were enrolled. All participants underwent brain magnetic resonance imaging scans and clinical scale assessments. QSM values were calculated and compared between TAO and HCs groups using a voxel-based analysis. A support vector machine (SVM) analysis was performed to evaluate the performance of QSM values in differentiating patients with TAO from HCs.

**Results:**

Compared with HCs, patients with TAO showed significantly increased QSM values in the bilateral caudate nucleus (CN), left thalamus (TH), left cuneus, left precuneus, right insula and right middle frontal gyrus. In TAO group, QSM values in left TH were positively correlated with Hamilton Depression Rating Scale (HDRS) scores (r = 0.414, p = 0.005). The QSM values in right CN were negatively correlated with Montreal Cognitive Assessment (MoCA) scores (r = -0.342, p = 0.021). Besides that, a nearly negative correlation was found between QSM values in left CN and MoCA scores (r = -0.286, p = 0.057). The SVM model showed a good performance in distinguishing patients with TAO from the HCs (area under the curve, 0.958; average accuracy, 90.1%).

**Conclusion:**

Patients with TAO had significantly increased iron deposition in brain regions corresponding to known visual, emotional and cognitive deficits. QSM values could serve as potential neuroimaging markers of TAO.

## 1 Introduction

Thyroid-associated ophthalmopathy (TAO), commonly known as Graves’ orbitopathy, is a progressing autoimmune disease associated with both severe physical and psychological disturbances (1). Commonly, patients with TAO are characterized by ophthalmic complaints, including upper eyelid retraction, proptosis, eyelids swelling, diplopia and impaired visual function (2). In addition to these typical physical manifestations, patients are also constantly obsessed with a series of emotional and psychiatric symptoms and even cognitive deficits, such as depression, anxiety, personality irregularities and poor concentration and memorization (3). These psychical and functional symptoms not only restrict patients’ daily activities like reading and driving, but also lead to dysfunctions in social roles and even increased risk of suicide (4, 5). Interestingly, a part of patients could experience emotional and psychiatric disturbances before the appearance of obvious ophthalmic symptoms (2). All these evidences suggested that TAO might be correlated with neuropsychic alterations rather than singly physical sufferings. Further, the negative impact on quality of life increases with the severity of the disease, which declines the socioeconomic status of the patients and even poses a significant burden on public health (6, 7).

In recent years, neuroimaging explorations focusing on structural and functional brain alternations in TAO have received more and more attentions. Prior studies based on T1-weighted images reported structural abnormalities in gray matters related to known visual, cognitive and emotional regulation (8, 9). Additionally, several resting-state functional magnetic resonance imaging (MRI) studies also reported altered brain activity in vision-, emotion- and cognition-related areas (10–12). However, most of the reported structural and functional brain abnormalities appear in the cortical regions, while few studies presented significant findings in deep gray matter regions. Utilization of advanced parameters or technologies that are more sensitive to deep gray matter disturbances are needed, in order to potentially delve and expand the existing understanding on the neurobiological mechanisms of TAO.

Iron, as an electron facilitator, is essential for maintaining the integrity of the neurochemical circuits (13). The distribution of brain iron concentration is not uniform, with the highest levels in deep gray matter regions, and also detectable in cortical regions (14). The dysregulation of iron homeostasis is confirmed to be related to neuropsychiatric disorders and cognitive impairments, and can be reflected through both blood and brain iron concentrations (13, 15). According to Xu et al.’s study on first-episode schizophrenia patients, reduced serum ferritin levels could be observed along with the decreased brain iron concentration in deep grey matter nuclei, verifying the internal association between the blood-brain iron (15). Coincidentally, it was reported that Graves’ hyperthyroidism could interfere with iron metabolism and elevate serum ferritin (16). In a recent study, several ferroptosis driver genes were found to exhibit relatively low expression levels in orbital tissues of TAO patients, indicating the abnormal iron metabolism in patients with TAO (17). However, whether the brain iron concentration of TAO patients exists abnormality remains unknown. The above findings also laid a foundation for us to explore the deep gray matter alterations of TAO patients using brain iron as a medium.

As a non-invasive MRI technique that can provide visualization and quantitative evaluation of brain iron, quantitative susceptibility mapping (QSM) can achieve both high sensitivity and specificity for detecting iron levels due to its good iron selectivity and reproducibility (18). Previously, the technique has already been applied to depict the abnormality of brain iron in several diseases, including amyotrophic lateral sclerosis, Parkinson’s disease and multiple sclerosis (19–21). Also, altered brain iron deposition presented by the technique was suggested to be correlated with the neuropsychiatric symptoms of these diseases (19–21). Therefore, given the current neuroimaging evidence and clinical findings, we hypothesized that patients with TAO would also have altered brain iron concentration that could be detected by QSM, especially in vision-, emotion- and cognition-related areas.

Here, we performed a voxelwise QSM analysis to verify our hypothesis and also evaluated the correlation between regional QSM values and the clinical variables. Besides that, considering that machine learning is increasingly applied to identify potential neuroimaging biomarkers, we applied a supervised machine learning approach that allows individual-level classification called support vector machine (SVM) (22) to detect whether QSM values could be used to differentiate patients with TAO from healthy controls (HCs).

## 2 Materials and methods

### 2.1 Subjects

This study was approved by the ethics committee of our hospital. The procedures used in this study adhere to the tenets of the Declaration of Helsinki. Written informed consent was obtained from all participants. Forty-eight patients with TAO (mean age, 45.42 ± 12.07 years; 27 females and 21 males) were recruited from the department of endocrinology in our hospital. Concurrently, 33 age-, gender- and education-matched HCs (mean age, 44.91 ± 13.46 years; 19 females and 14 males) were recruited from the local community. All the patients stayed in euthyroid status (reference ranges: FT3, 3.10 - 6.80 pmol/L; FT4, 12.00 - 22.00 pmol/L; TSH, 0.27 - 4.20 mIU/L) for at least 3 months before MRI scan. Forty-three among the patients were treated with drug therapy, 3 were treated with surgical therapy, and 2 were treated with isotope therapy. The exclusion criteria were as follows: (1) any evidence for other ocular pathologies (inflammation, amblyopia, glaucoma, cataracts, etc.); (2) history of eye surgery; (3) history of neurological or psychiatric disorders (e.g. depression, schizophrenia, bipolar disorder); (4) contraindications for MRI examination.

### 2.2 Clinical assessment

The clinical diagnosis of TAO was made by an experienced endocrinologist. Disease duration of TAO was defined from the onset of TAO-related symptoms. Disease activity was assessed according to the modified seven-point Mourits’ clinical activity score (CAS) (23). All participants underwent visual acuity measurement, and the numerical value of the worse eye was recorded.

Authoritative questionnaires about life quality and neuropsychological assessments were conducted before the MRI examination. The Quality of Life (QoL) questionnaire, which contains two subscales respectively for visual functioning and appearance, was completed on each patient. The degree of anxiety and depression were assessed in all subjects by 14-item Hamilton Anxiety Rating Scale (HARS) and 17-item Hamilton Depression Rating Scale (HDRS), respectively. Montreal Cognitive Assessment (MoCA) was used to assess cognitive function in all participants.

### 2.3 Imaging acquisition

All the examinations were performed by a 3.0 T MRI unit (Magnetom Skyra; Siemens Healthcare, Erlangen, Germany) with a 20-channel head and neck coil. All the participants were instructed to lie still in the supine position. Foam padding and earplugs were provided to reduce head movement and noise during scanning. A multi-gradient echo sequence with five echoes was used to obtain both phase and magnitude images using the following parameters: repetition time (TR) = 51 ms; five equidistant echo time (TE) between 9.34 and 45.00 ms; flip angle = 20°; field of view (FOV) = 218 × 240 mm^2^; matrix size = 363 × 416; slice thickness = 2.2 mm without gap; voxel size = 0.6 × 0.6 × 2.2 mm^3^; slice number = 56; scan time = 5 min. In addition, the three dimension structural T1-weighted images were acquired using magnetization-prepared rapid gradient echo sequence with the following parameters: TR = 1900 ms; TE = 2.45 ms; flip angle = 9°; FOV = 256 × 256 mm^2^; matrix size = 256 × 256; slice thickness = 1 mm without gap; voxel size = 1 × 1 × 1 mm^3^; slice number = 176; scan time = 4 min and 18 s.

### 2.4 Voxel-based QSM analyses

The STI Suite toolbox version 3.0 (https://people.eecs.berkeley.edu/~chunlei.liu/software.html) embedded in MATLAB R2013b (Mathworks, Natick, MA, USA) was used for the reconstruction of QSM images, including phase unwrapping, background field removal and dipole inversion. Firstly, the unwrapped phase images were created by a Laplacian-based method. Secondly, the variable-kernel sophisticated harmonic artifact reduction for phase data (V-SHARP) algorithm was used to remove the background field (because of the air-tissue interface). Finally, the susceptibility map was obtained using the streaking artifacts reduction in QSM (STAR-QSM) algorithm.

Further, the functional MRI of the brain (FMRIB) software library (FSL version 5.0, http://www.fmrib.ox.ac.uk/fsl) was used for spatial normalization. The first echo magnitude images of each subject were coregistered to the 3D T1 anatomical images. Concurrently, T1 images were normalized to Montreal Neurological Institute space. Thereafter, all the QSM images were transformed to MNI space using the generated warp transformation parameters and smoothed by an 8-mm full width at half maximum Gaussian kernel.

For the QSM values [parts per billion (ppb)], voxel-based whole-brain analyses were performed using Statistical Parametric Mapping (SPM) 12. A two-sample t-test with age, gender and years of education as confounding covariates was performed to assess the differences between groups. Statistical significance was determined using the family-wise error (FWE) correction for multiple comparisons (voxel-level significance: p < 0.001; cluster-level significance: p < 0.05). The mean QSM values in each significant cluster were extracted for each patient. Brain regions corresponding to the significant clusters were identified in reference to the Automated Anatomical Labeling (AAL) atlas. The mean QSM values in each brain region were also extracted for subsequent analyses.

### 2.5 Statistical analyses

SPSS software (Version 25.0, Inc., Chicago, IL, USA) was used for statistical analyses. Shapiro-Wilk test was used to assess the normality of the quantitative data. If normally distributed, the quantitative data would be presented as mean ± standard deviation. Otherwise, expressed as median (interquartile range). The continuous variables of the TAO and HC groups were compared using two-sample t-tests (for data with normal distribution) or Mann-Whitney U tests (for data with skewed distribution). The categorical variables of the two groups were compared using Chi-square tests. A value of p < 0.05 was considered statistically significant. Partial correlation analyses were performed to evaluate the relationships between QSM values and clinical variables in TAO groups after controlling the effect of age, gender and years of education. The statistical threshold was set at uncorrected p < 0.05 due to the exploratory nature of these analyses.

### 2.6 Support vector machine analyses

SVM analysis was performed using the LIBSVM software to detect whether QSM value could be used as a potential neuroimaging biomarker to differentiate patients with TAO from HCs. To reduce the risk of overfitting and directly extract the feature weights, a linear kernel SVM was used to perform the classifier training. The performance of the classifier was evaluated using a “leave-one-out” cross-validation approach. The cross-validated performance of the SVM model was evaluated by receiver operating characteristic (ROC) curve analysis. The significance of classification accuracy was validated using a non-parametric permutation test with 5000 permutations. All detailed steps of SVM analysis could be referred to a previous study (10).

### 2.7 Validation of susceptibility data

To validate the voxel-based QSM analyses, we correlated the QSM values with iron concentrations derived from postmortem histology. QSM values of six subcortical regions, including globus pallidus (GP), red nucleus (RN), substantia nigra (SN), putamen (PUT), caudate nucleus (CN) and thalamus (TH), were extracted from the normalized QSM images of all the HCs. The values were averaged across the bilateral cerebral hemispheres. The postmortem brain iron concentrations in these subcortical regions were reported in the previous study (24).

## 3 Results

### 3.1 Demographic and clinical data

Detailed demographic and clinical characteristics of all subjects were summarized in **Table 1**. There was no significant difference in age (p = 0.923), gender (p = 0.906) and years of education (p = 0.996) between TAO and HC group. Compared with the HC group, patients with TAO had significantly decreased visual acuity (p = 0.002). Moreover, patients had significantly higher total scores of HDRS and HARS (both p < 0.001), as well as lower total scores of MoCA (p < 0.001) than the HC group.

**Table 1:** Demographic and clinical characteristics of patients with TAO and HCs.

Variable	TAO (n = 48)	HC (n = 33)	p value
**Age (year)**	45.42 ± 12.07	44.91 ± 13.46	0.923
**Gender (female/male)**	27/21	19/14	0.906
**Education level (years)**	11.48 ± 3.21	11.39 ± 3.63	0.996
**Visual acuity**	0.86 ± 0.19	0.98 ± 0.10	0.002
Disease duration (months)			
** TAO**	17.83 ± 21.29	–	
** Graves’ disease**	35.10 ± 52.84	–	
**TRAb (IU/L)**	6.59 ± 10.09		
**CAS**	2.85 ± 1.44	–	
Total score of QoL			
** Visual functioning**	60.57 ± 22.86	–	
** Appearance**	64.96 ± 20.02	–	
**Total score of HDRS**	12.04 ± 6.11	2.30 ± 1.57	< 0.001
**Total score of HARS**	14.13 ± 5.27	2.85 ± 1.75	< 0.001
**Total score of MoCA**	26.71 ± 1.79	28.97 ± 0.98	< 0.001

Data are presented as mean ± standard deviation (SD).

TAO, thyroid-associated ophthalmopathy; HC, healthy control; TRAb, thyrotropin receptor antibody; CAS, clinical activity score; QoL, quality of life; HDRS, Hamilton Depression Rating Scale; HARS, Hamilton Anxiety Rating Scale; MoCA, Montreal Cognitive Assessment.

### 3.2 Voxel-based QSM analyses

**Figure 1:**
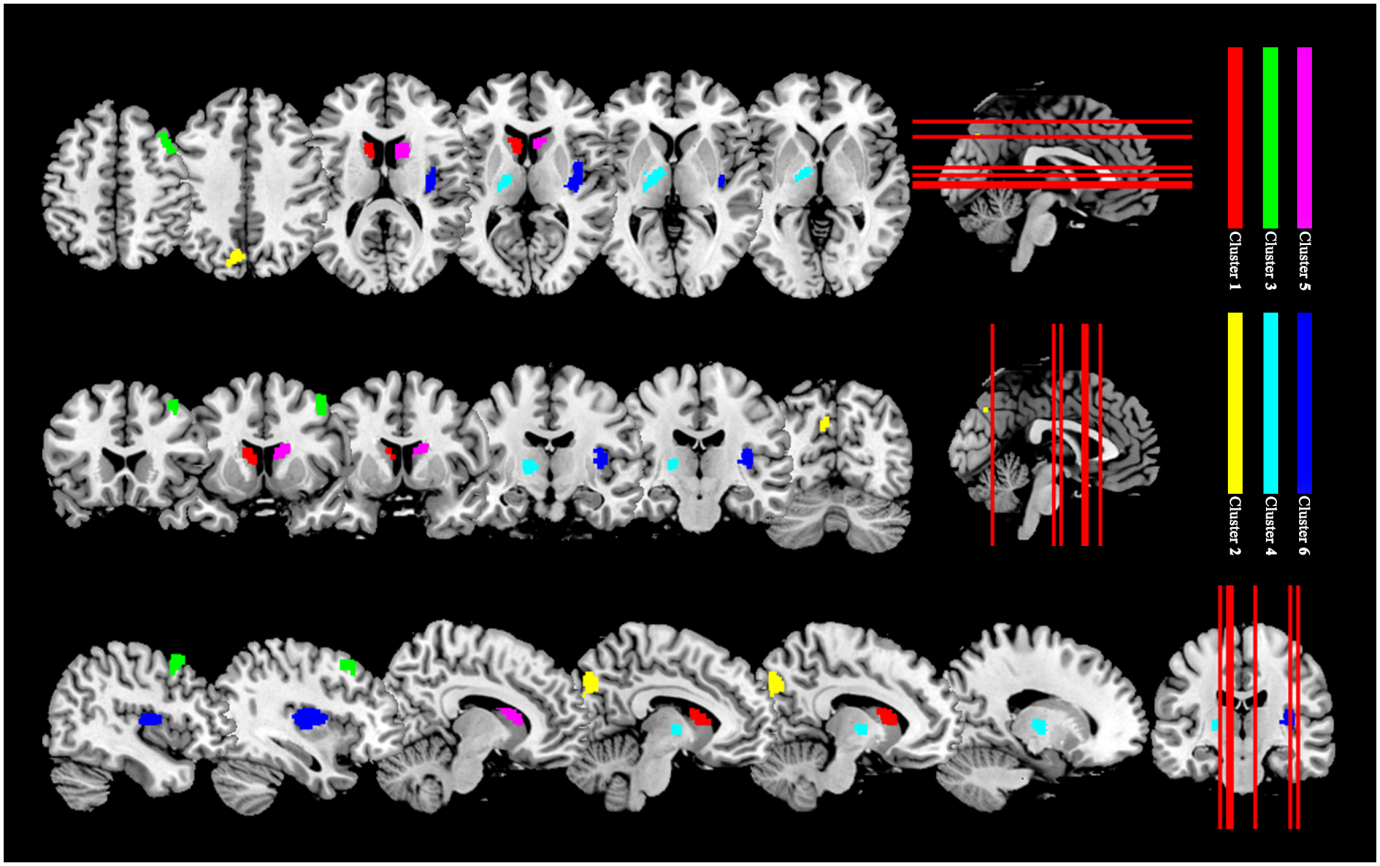
Clusters with significantly increased QSM values in patients with TAO than HCs (p < 0.05, cluster-level FWE corrected). Cluster 1 (red): left caudate nucleus; Cluster 2 (yellow): left precuneus and left cuneus; Cluster 3 (green): right middle frontal gyrus; Cluster 4 (light blue): left thalamus; Cluster 5 (magenta): right caudate nucleus; Cluster 6 (dark blue): right insula. QSM, quantitative susceptibility mapping; TAO, thyroid-associated ophthalmopathy; HC, healthy control; FWE, family-wise error.

Six clusters showed increased QSM values in TAO group than HC group (p < 0.05, cluster-level FWE corrected). The clusters were located in bilateral cerebral hemispheres, including bilateral CN, left TH, left cuneus (CUN), left precuneus (PCU), right insula (INS) and right middle frontal gyrus (MFG). Detailed information for significant clusters is shown in **Table 2** and **Figure 1**.

**Table 2:** Brain areas with significantly altered QSM values between groups (p < 0.05, cluster-level FWE corrected).

No	Voxels	PV_X	PV_Y	PV_Z	AAL	Volume (mm^3^)	*T* value	p value
**1**	167	-8	14	10	CN_L	1280	7.62	0.042
**2**	167	-10	-74	38	PCU_L	776	4.86	0.042
					CUN_L	408		
**3**	168	40	16	52	MFG_R	1272	4.33	0.041
**4**	179	-18	-12	4	TH_L	1136	6.44	0.032
**5**	205	14	10	16	CN_R	1376	5.61	0.018
**6**	304	36	-20	10	INS_R	1928	5.53	0.003

QSM, quantitative susceptibility mapping; FWE, family-wise error; PV, peak voxel; X, Y, Z, coordinates in the Montreal Neurologic Institute (MNI) atlas; AAL, Automated Anatomical Labeling atlas; CN, caudate nucleus; PCU, precuneus; CUN, cuneus; MFG, middle frontal gyrus; TH, thalamus; INS, insula; L, left; R, right.

### 3.3 Correlation analyses

**Figure 2:**
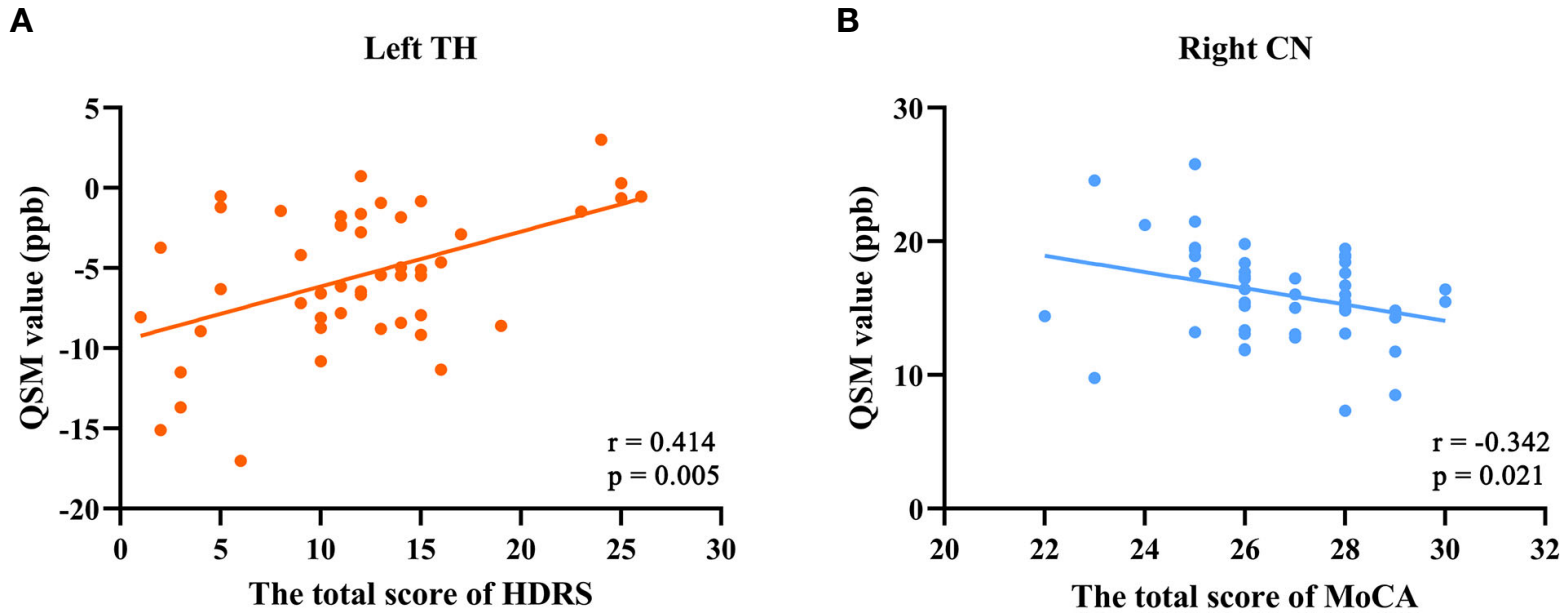
Correlation analyses results between QSM values and clinical variables. **(A)** QSM values in left TH were positively correlated with the total scores of HDRS (r = 0.414, p = 0.005). **(B)** QSM values in right CN were negatively correlated with the total scores of MoCA (r = -0.342, p = 0.021). Age, gender and years of education were included as covariates. QSM, quantitative susceptibility mapping; TH, thalamus; CN, caudate nucleus; HDRS, Hamilton Depression Rating Scale; MoCA, Montreal Cognitive Assessment.

In patients with TAO, QSM values in left TH were positively correlated with the scores of HDRS (r = 0.414, p = 0.005) (**Figure 2A**). The QSM values in right CN were negatively correlated with the scores of MoCA (r = -0.342, p = 0.021) (**Figure 2B**). Additionally, a nearly negative correlation was also found between QSM values in the left CN and the scores of MoCA (r = -0.286, p = 0.057). No significant correlation was found between QSM values and other clinical parameters including CAS, QoL scores, HARS scores, visual acuity and disease duration.

### 3.4 Support vector machine analyses

**Figure 3:**
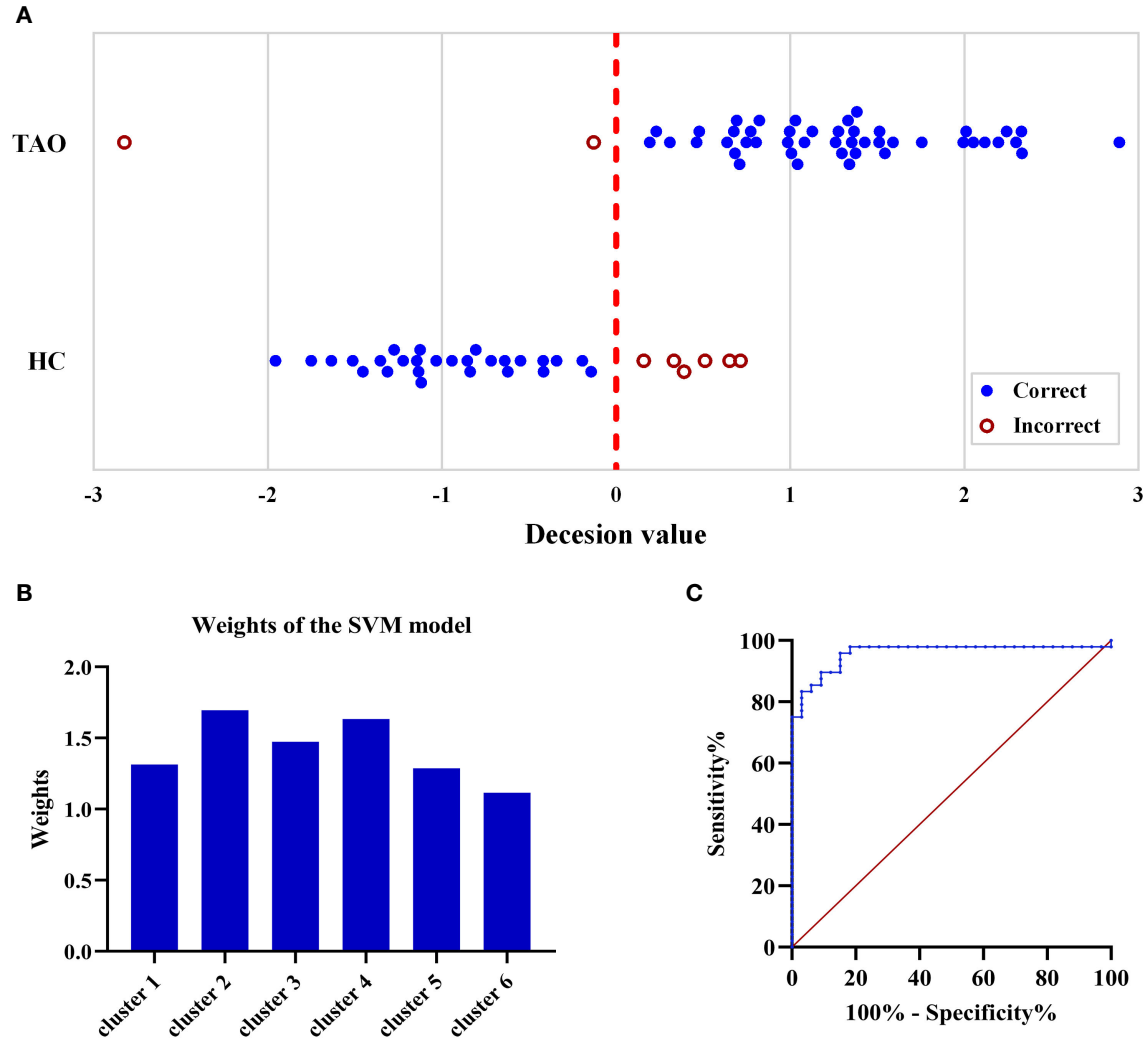
Illustration of the SVM classification results. **(A)** The dark blue closed circle represent the correct classification, while the dark red open circle represent the incorrect classification. The final accuracy of the SVM model was 90.1% (73/81). **(B)** The weight of clusters 1 to 6 in classifying the two groups were 1.315, 1.696, 1.474, 1.635, 1.288 and 1.115, respectively. **(C)** The ROC curve showed that the SVM model had satisfactory performance in distinguishing patients with TAO and HCs, with an AUC of 0.958 (p < 0.001). SVM, support vector machine; TAO, thyroid-associated ophthalmopathy; HC, healthy control; ROC, receiver operating characteristic; AUC, area under the curve.

The average accuracy of the SVM model was 90.1% (p < 0.001, permutation approach with 5000 permutations) (**Figure 3A**). The weight of clusters 1 to 6 were 1.315, 1.696, 1.474, 1.635, 1.288 and 1.115, respectively (**Figure 3B**). The SVM model had an area under the curve (AUC) of 0.958, indicating a good performance to distinguish patients with TAO from HCs (**Figure 3C**). The sensitivity and specificity were 89.6% and 90.9%, respectively.

### 3.5 Validation of susceptibility data

**Figure 4:**
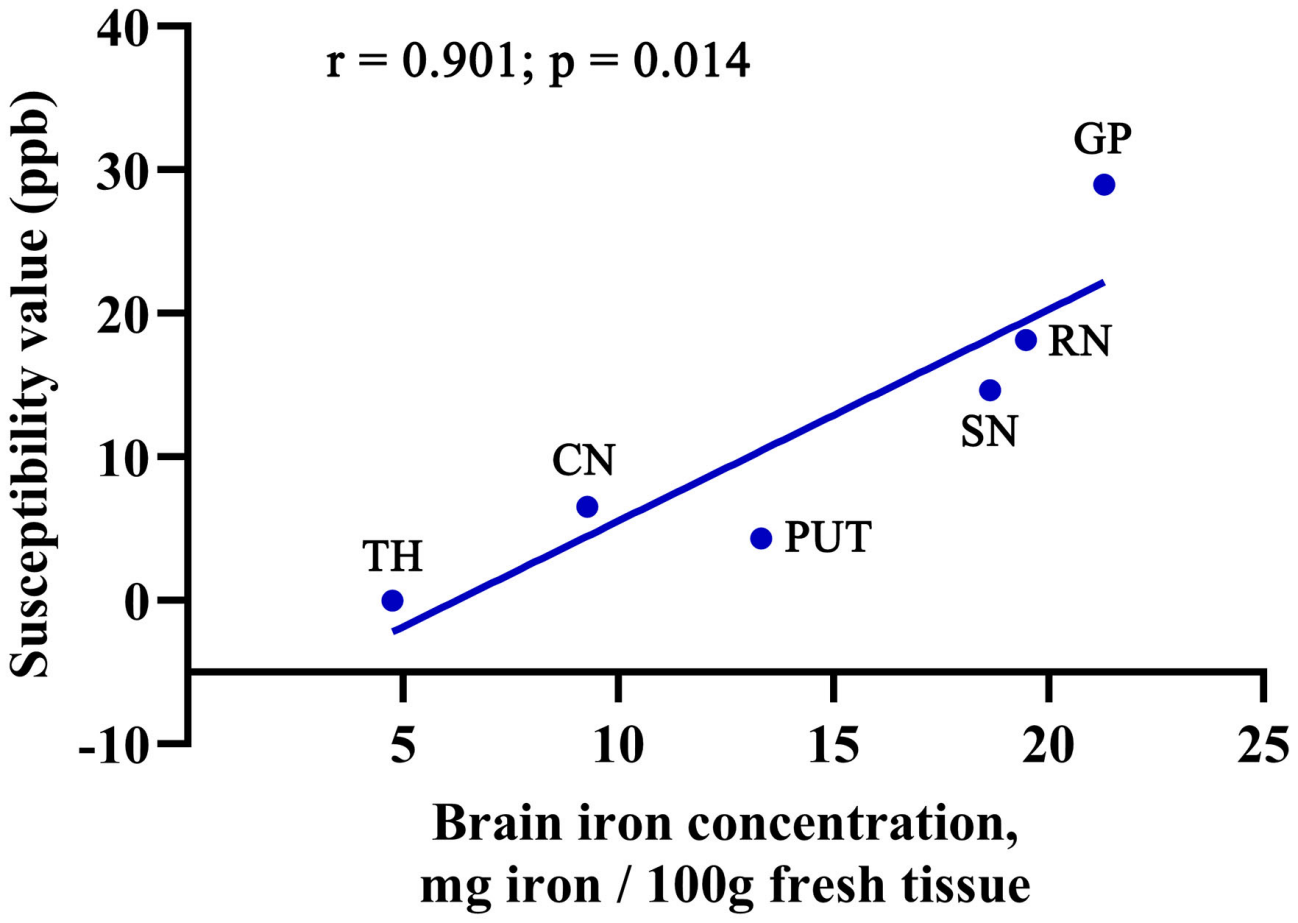
Validation of QSM values with histologically measured brain iron. In the 33 HCs, the mean QSM values of GP, RN, SN, PUT, CN and TH were 28.98, 18.15, 14.65, 4.33, 6.52 and -0.04 ppb, respectively. The postmortem brain iron concentrations in these subcortical regions reported by Hallgren and Sourander were 21.30, 19.48, 18.64, 13.32, 9.28 and 4.76 mg per 100g of fresh weight, respectively. The QSM values of GP, RN, SN, PUT, CN and TH were positively correlated with the brain iron concentrations reported by Hallgren and Sourander in these subcortical regions (r = 0.901, p = 0.014). QSM, quantitative susceptibility mapping; GP, globus pallidus; RN, red nucleus; SN, substantia nigra; PUT, putamen; CN, caudate nucleus; TH, thalamus.

In the 33 HCs, the mean QSM values of GP, RN, SN, PUT, CN and TH were 28.98, 18.15, 14.65, 4.33, 6.52 and -0.04 ppb, respectively. The postmortem brain iron concentrations in these subcortical regions reported by Hallgren and Sourander were 21.30, 19.48, 18.64, 13.32, 9.28 and 4.76 mg per 100g of fresh weight, respectively. A close positive correlation was found between the QSM values in the present study and the reported postmortem iron concentrations in these brain regions (r = 0.901, p = 0.014) (**Figure 4**).

## 4 Discussion

We applied QSM technique integrated with machine learning method to investigate brain alterations in patients with TAO. Our current exploratory cross-sectional study had three main findings. First, patients with TAO had significantly increased QSM values in both deep gray matter regions and cortical regions. Second, significant correlations were presented between increased QSM values in some of these regions and clinical performances in the TAO group. Third, the SVM model achieved satisfactory accuracy and efficacy in distinguishing patients with TAO and the HCs. The preliminarily findings provided novel evidence to interpret the clinical-psychological symptoms of TAO from the perspective of iron deposition, and expanded the understanding of the underlying neural mechanism of the disease.

In this study, increased QSM values in bilateral CN were observed in the TAO group. As part of the fronto-striatal circuit, CN is thought to play an essential role in cognitive processes (25). Destruction of the CN may cause cognitive impairment such as slowed information processing, poor short- and long-term memory and decreased executive function (13, 25). Previously, several ROI-based QSM studies reported that increased QSM values in CN could be detected in diseases such as hypertension with mild cognitive impairment, cerebral autosomal dominant arteriopathy and Parkinson, and QSM values were negatively correlated with the total scores of MoCA (13, 26, 27). Another voxel-based QSM analysis also found that patients with Parkinson’s disease had increased QSM values in CN, suggested to be associated with the excessive iron deposition and cognitive decline (28). In line with these findings, our current study regarding TAO also detected increased QSM values in bilateral CN, indicating the increased iron deposition in site. Together with the negative correlations between QSM values and the total scores of MoCA, it could further confirm that excessive iron deposition in CN would be related to the abnormality of cognitive regulation in TAO.

Abnormal iron deposition in left TH was another important finding in the deep gray matter regions in our study. TH, a complex sensory information node between the cerebral cortex and subcortical regions, is closely related to emotion, arousal and memory (29). Iron deposition in TH might cause dysfunction of local neurons and neurotransmitters and finally lead to depressive symptoms (30). Abnormal connections in thalamus-cortex areas have been found in patients with depression (31). Meanwhile, a previous study using QSM analysis reported that the increased iron accumulation in the left TH was associated with the severity of depression based on the total score of HDRS (32). Similarly, in this study, we also detected increased QSM values in left TH in TAO. Combining their positive correlations with the total scores of HDRS, we could suggest that abnormal iron deposition in TH might be relevant to emotional dysfunctions in patients with TAO.

In addition to the deep gray matter alternations in bilateral CN and left TH, increased QSM values were also found in several cortical regions related to cognition, emotion and vision. INS is located in the depths of the lateral sulcus and is involved in high-level cognitive functions and socio-emotional processing (33). The dorsal anterior INS has connections with anterior cingulate, frontal and parietal areas is responsible for cognitive control processes and the ventral anterior INS with connections to limbic areas is responsible for affective processes (33). Increased amplitude of low-frequency fluctuation values of INS was found in TAO in a resting-state functional MRI study, which was attributed to the altered emotional processing (34). PCU is the posteromedial portion of the parietal lobe and plays an important role in various highly integrated tasks, such as visuo-spatial imagery, recollection and memory (35). Moreover, PCU is a brain region implicated in the default mode network, which also plays an important role in self-consciousness (35). Luo et al. found that the changes of gray matter volume in PCU were associated with affective and cognitive dysfunctions in active TAO (36). Decreased voxel-mirrored homotopic connectivity of PCU was also found in TAO in a resting-state functional MRI study (10). CUN is a wedge-shaped area of the occipital lobe, which is well known as an important part of visual path. It is reported to interact with primary visual cortex V1 to modify visual information and is related to visual field, spacial location and eye movement reflex (37, 38). A prior study using surface-based morphometry detected decreased cortical complexity in CUN, suggesting to be associated with the lower half visual field defect and visual impairment in TAO (9). The MFG is an important part of the salience network, which is responsible for complicated processes of cognition, including concentration, memory and decision-making (39). Of note, as a part of dorsolateral prefrontal cortex, the MFG is also thought to be involved in spatial perception and manipulating the visual information (40). Chen et al. found that the regional homogeneity values increased significantly in MFG in TAO, and had a strong correlation with the total score of MoCA (41). Similar to these structural and functional neuroimaging findings, the currently detected higher QSM values in the above brain regions might also make effect on cognition-, emotion- and vision-related dysfunctions in TAO to some extent, in spite of the nonsignificant correlations with clinical indexes.

For the sake of further application to the individual level and improved translational potential in a clinical setting, an SVM analysis was performed after the inter-group QSM analysis. We tested the QSM values as features to differentiate patients with TAO and HCs through a linear SVM classifier. The SVM model achieved a satisfactory AUC of 0.958 with an average accuracy of 90.1%. This finding built the confidence that brain QSM value could be used as a potential neuroimaging biomarker for individualized differentiation between patients with TAO and HCs. The current study could also act as an essential pillar for more profound kinetics researches on the mechanism of abnormal iron deposition in patients with TAO.

Several limitations should be acknowledged in our study. First, the sample size is relatively small due to the challenge of recruiting patients with euthyroid status. Further studies with a lager sample size would strengthen the statistical power and verify the present findings. Second, we only enrolled TAO patients in this study. Further analysis of the alteration of iron deposition in patients with Graves’ disease without TAO may provide additional insights into the disease. Third, current study focused on the susceptibility in the gray matter, while the susceptibility related to the diamagnetic effect of myelin in white matter could also contribute to the understanding of TAO. Fourth, this study only explored the relationship between clinical scale scores and the abnormal iron deposition. Hematology parameters such as serum ferritin also need to be collected in the further study. Last, the cross-sectional nature of our study may limit the profundity of findings. The quantitation of QSM enables longitudinal researches to investigate the changes of brain iron status during the process of TAO, including the follow-up after treatment.

In conclusion, our study indicated that abnormal QSM values were observed in both deep gray matter regions and cortical regions in patients with TAO, and were statistically correlated with clinical scale scores, reflecting potential cognitive, emotional, and visual dysfunctions. These findings provided new insight into the understanding of TAO, especially in the neurobiological aspect. The QSM values could serve as neuroimaging markers to differentiate patients with TAO from healthy subjects.

## Data availability statement

The raw data supporting the conclusions of this article will be made available by the authors, without undue reservation.

## Ethics statement

The studies involving humans were approved by the Ethics Committee of the First Affiliated Hospital of Nanjing Medical University. The studies were conducted in accordance with the local legislation and institutional requirements. The participants provided their written informed consent to participate in this study.

## Author contributions

HH: Data curation, Formal Analysis, Funding acquisition, Methodology, Writing – original draft. JZ: Data curation, Formal Analysis, Software, Validation, Writing – original draft. WF: Formal Analysis, Writing – original draft. H-HC: Resources, Writing – review & editing. W-HJ: Data curation, Formal Analysis, Writing – original draft. X-YP: Data curation, Formal Analysis, Writing – original draft. W-HG: Software, Writing – original draft, Writing – review & editing. F-YW: Conceptualization, Project administration, Resources, Supervision, Writing – review & editing. X-QX: Conceptualization, Funding acquisition, Project administration, Resources, Writing – review & editing.
